# Deoxypodophyllotoxin suppresses tumor vasculature in HUVECs by promoting cytoskeleton remodeling through LKB1-AMPK dependent Rho A activation

**DOI:** 10.18632/oncotarget.4985

**Published:** 2015-07-22

**Authors:** Yurong Wang, Bin Wang, Mounia Guerram, Li Sun, Wei Shi, Chongchong Tian, Xiong Zhu, Zhenzhou Jiang, Luyong Zhang

**Affiliations:** ^1^ Jiangsu Key Laboratory of Drug Screening and Jiangsu Center for Pharmacodynamics Research and Evaluation, China Pharmaceutical University, Nanjing, P.R. China; ^2^ Chia Tai Tianqing Pharmaceutical Group Co., Ltd, Nanjing, P.R. China; ^3^ Medical and Chemical Institute, China Pharmaceutical University, Nanjing, P.R. China; ^4^ Jiangsu Key Laboratory of TCM Evaluation and Translational Research, China Pharmaceutical University, Nanjing, P.R. China; ^5^ State Key Laboratory of Natural Medicines, China Pharmaceutical University, Nanjing, P.R. China

**Keywords:** deoxypodophyllotoxin, tumor vasculature, cytoskeleton remodeling, Rho A, AMP-activated protein kinase

## Abstract

Angiogenesis plays a critical role in the growth and metastasis of tumors, which makes it an attractive target for anti-tumor drug development. Deoxypodophyllotoxin (DPT), a natural product isolated from *Anthriscus sylvestris*, inhibits cell proliferation and migration in various cancer cell types. Our previous studies indicate that DPT possesses both anti-angiogenic and vascular-disrupting activities. Although the RhoA/ RhoA kinase (ROCK) signaling pathway is implicated in DPT-stimulated cytoskeleton remodeling and tumor vasculature suppressing, the detailed mechanisms by which DPT mediates these effects are poorly understood. In the current study, we found that DPT promotes cytoskeleton remodeling in human umbilical vein endothelial cells (HUVECs) via stimulation of AMP-activated protein kinase (AMPK) and that this effect is abolished by either treatment with a selective AMPK inhibitor or knockdown. Moreover, the cellular levels of LKB1, a kinase upstream of AMPK, were enhanced following DPT exposure. DPT-induced activation of AMPK in tumor vasculature effect was also verified by transgenic zebrafish (VEGFR2:GFP), Matrigel plug assay, and xenograft model in nude mice. The present findings may lay the groundwork for a novel therapeutic approach in treating cancer.

## INTRODUCTION

Targeting tumor vasculature has been increasingly recognized as an alternative strategy to control the growth and metastases of solid tumors [1]. Vascular targeting agents (VTAs) are classified as angiogenesis inhibitors (AIs) and vascular disrupting agents (VDAs) [2]. AIs have been widely used as clinical cancer treatments. However, they are only efficient on small tumors and their effects on existing blood vessels within well-established tumors are not potent. VDAs induce a rapid and selective shutdown of established tumor vasculature by disrupting the tumor endothelium and exert significant effects on large experimental tumors. However, VDAs have a limited effect on the thin rim of the tumor periphery, where angiogenesis most vigorously occurs, and few compounds exhibit dual effects on tumor vasculature [3–6]. Reports regarding these compounds are rare, and the exact mechanism underlying the protective actions of these compounds remains unclear.

Deoxypodophyllotoxin (DPT), isolated from *Anthriscus sylvestris*, exerts anti-proliferative and pro-apoptotic effects against a broad variety of tumors both *in vitro* and *in vivo* [7–10]. DPT promotes cytoskeletal remodeling and possesses both anti-angiogenic and vascular-disrupting activities. Although the Rho A / Rho A kinase (ROCK) pathway has been implicated in these effects [11], the underlying mechanisms behind these activities are poorly understood.

One emerging concept regarding the mechanism of tumorigenesis is that metabolic changes not only participate in but also critically impact the excessive proliferation of tumor cells [12, 13]. In particular, AMP-activated protein kinase (AMPK), a highly conserved protein kinase regulating mammalian metabolism, has been increasingly recognized as a central regulator in the physiological development of tumor cells [14]. AMPK activators suppress the incidence and development of tumors in some model systems [15, 16]. However, AMPK activation is emerging as a master molecular switch that promotes cytoskeletal remodeling in various cell types [17, 18]. To the best of our knowledge, no reports regarding the effects of AMPK-induced cytoskeletal remodeling on tumor vasculature have been previously published.

In the present study, we investigated the potential role and underlying mechanisms of DPT as an effective VTA in the treatment of tumors. We found that DPT promotes cytoskeletal remodeling in human umbilical vein endothelial cells (HUVECs) via AMPK stimulation. We further demonstrated that LKB1-dependent AMPK activation is an unrecognized and important mechanism by which DPT promotes cytoskeletal remodeling.

## RESULTS

### DPT-induced cell contraction is accompanied by actin polymerization and microtubule depolymerization

**Figure 1:**
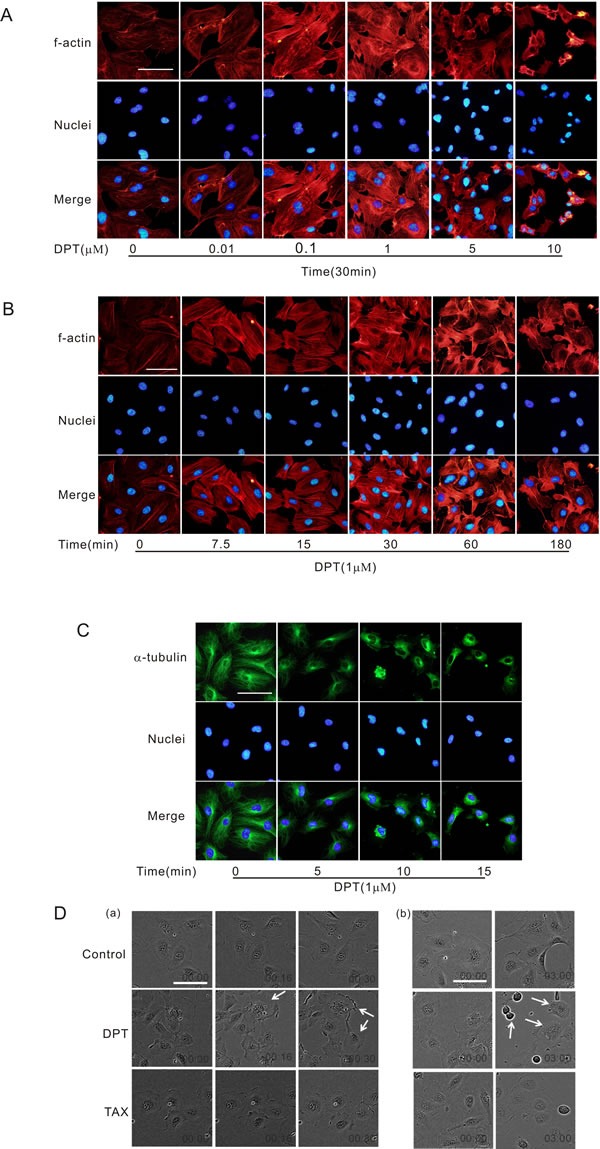
DPT-induced cell contraction was accompanied by actin polymerization and microtubule depolymerization **A.** Visualization of actin polymerization in HUVECs. Cells were treated with different concentrations of Deoxypodophyllotoxin (DPT) for 30 min. **B.** Visualization of actin polymerization in HUVECs. Cells were treated with 1 μM DPT for the indicated times. **C.** Visualization of tubulin depolymerization in HUVECs. Cells were treated with 1 μM DPT for the indicated times. **D.** Live cell microscopy of HUVECs. Cells were treated with 1 μM DPT or taxol (TAX) for different times. Scale bar: 50 μm.

It has been suggested that changes in endothelial cell morphology may be associated with vascular targeting activities. Cell contraction and cytoskeletal remodeling may cause obstruction of cell migration and increase in vascular resistance. DPT promoted actin stress fiber formation in HUVECs in a dose- and time-dependent manner (Figure 1A and 1B). DPT also disrupted microtubule dynamics in HUVECs in a time-dependent manner (Figure 1C). Moreover, rapid contractions were observed in DPT-treated cells using live-cell microscopy. This response was evident from the retraction of the cell margins, and the cell membrane integrity was eradicated after 3 h (white arrows, Figure 1D). However, the loss of membrane integrity was not observed in HUVECs treated with taxol, the natural microtubule stabilizer used as control (Figure 1D). Meanwhile, we confirmed that DPT didn't induce apoptosis at the concentrations used for this study (Supplementary Figure 1). These data indicated that cytoskeletal remodeling accompanied by actin polymerization and microtubule depolymerization account for DPT's ability to suppress tumor vasculature.

### RhoA/ROCK signaling pathway is involved in DPT-induced cytoskeletal remodeling and its relationship with actin and tubulin

**Figure 2:**
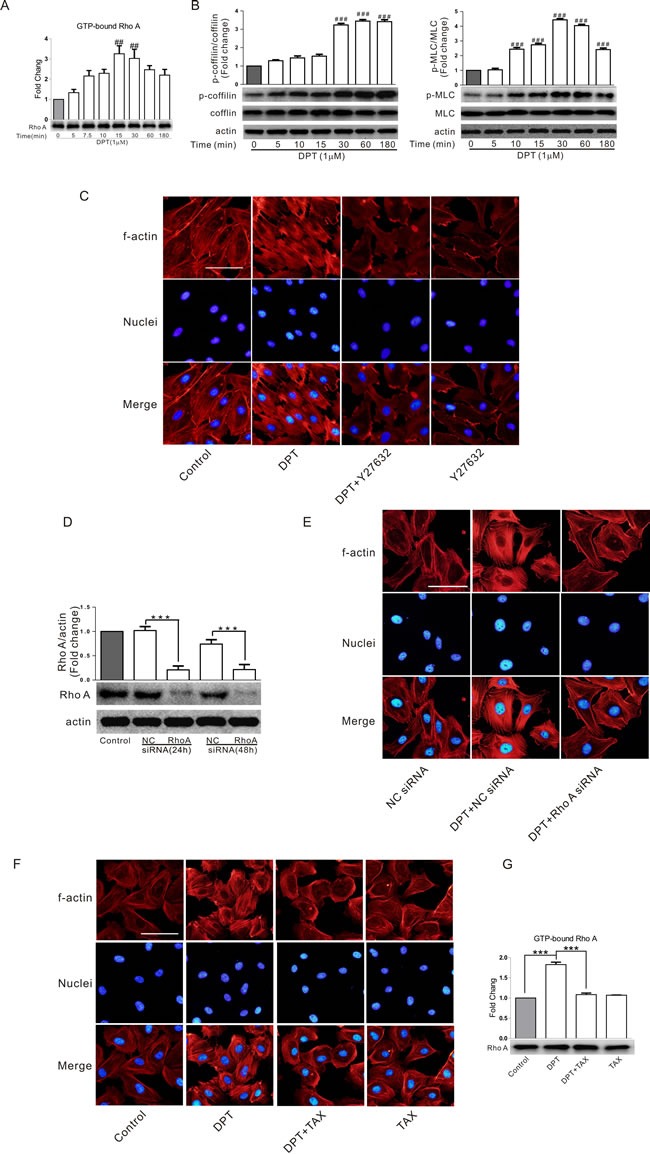
RhoA/ROCK signaling pathway was involved in DPT-induced cytoskeletal remodeling and its relationship with actin and tubulin **A.** G-LISA measurement of protein concentrations of GTP-bound RhoA in cell lysates of HUVECs. Cells were treated with 1 μΜ Deoxypodophyllotoxin (DPT) for the indicated times. **B.** Western blot measurement of the expression of cofilin and MLC phosphorylation in HUVECs. Cells were treated with 1 μM DPT for the indicated times. **C.** Visualization of actin polymerization in HUVECs. Cells were pretreated with 10 μM Y27632 for 30 min, followed by treated with 1 μM DPT for 30 min. **D.** Western blot measurement of expression of RhoA in HUVECs. Cells were transfected with 10 nM RhoA siRNA or control siRNA (NC siRNA) for different times. **E.** Visualization of actin polymerizationin in HUVECs. Cells were pretreated with 10 nM Rho A siRNA or NC siRNA for 24 h, followed by treated with 1 μM DPT for 30 min. **F.** Visualization of actin polymerization in HUVECs. Cells were pretreated with 1μM taxol (TAX) for 30 min, followed by treated with 1 μM DPT for 30 min. **G.** G-LISA measurement of protein concentrations of GTP-bound RhoA in cell lysates of HUVECs. Cells were pretreated with 1μΜ taxol for 30 min, followed by treated with 1 μM DPT for 30 min. Results are means ± SEM of at least three independent experiments. *** *p* < 0.001; ##*p* < 0.01, ###*p* < 0.001, compared with 0 h. Scale bar: 50 μm.

Rho/Rho kinase signalling pathway is a critical regulator of cytoskeleton and cell behaviour. To confirm the mechanism by which DPT induced cytoskeletal remodeling, we examined RhoA activity in HUVECs lysates using a commercial G-LISA kit. RhoA activation peaked 15 min after DPT exposure (1 μM) and then slightly declined (Figure 2A). DPT also increased the phosphorylation of cytoskeletal proteins, especially regulatory coﬁlin and myosin light chains (MLCs) (Figure 2B), two key elements involved in actin cytoskeletal contraction and polymerization. The ROCK inhibitor Y27632 prevented DPT-induced stress fiber formation (Figure 2C), suggesting that activation of RhoA/ROCK signaling pathway is upstream of actin polymerization. Consistent with these results, RhoA knockdown by siRNA markedly decreased stress fiber formation (Figure 2D and 2E). Taxol, a well-known microtubule stabilizer, completely inhibited DPT-induced RhoA activation and actin stress fiber formation (Figure 2F and 2G), indicating that microtubule depolymerization triggered activation of RhoA/ROCK signaling pathway.

### AMPK activation is involved in DPT-mediated cytoskeletal remodeling in HUVECs

**Figure 3:**
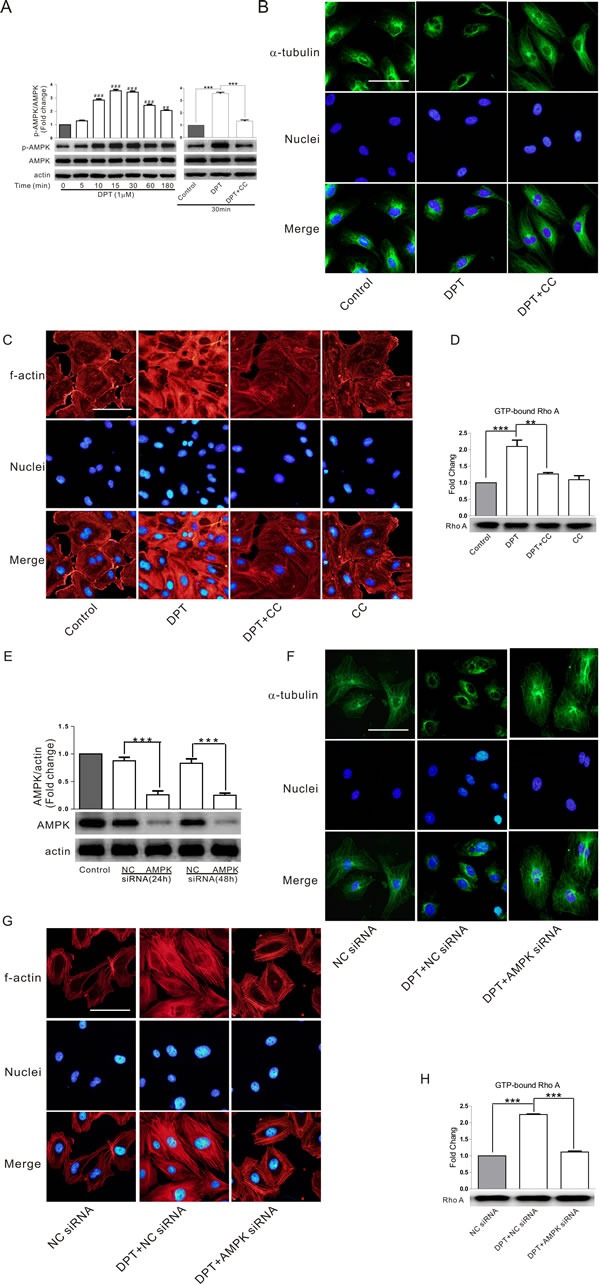
AMPK activation was involved in DPT-mediated cytoskeleton remodeling in HUVECs **A.** Western blot measurement of the expression of AMPK phosphorylation in HUVECs. Cells were treated with 1 μM Deoxypodophyllotoxin (DPT) for different times, or pretreated with 10 μM AMPK inhibitor Compound C (CC) for 2h, followed by treatment with1 μM DPT for the indicated times. **B.** and **C.** Visualization of tubulin depolymerization and actin polymerization in HUVECs. Cells were pretreated with 10 μM CC for 2 h, followed by treated with 1μM DPT for 30 min. **D.** G-LISA measurement of protein concentrations of GTP-bound RhoA in cell lysates of HUVECs. Cells were pretreated with 10 μM CC for 2 h, followed by treated with 1 μM DPT for 30 min. **E.** Western blot measurement of expression of AMPK in HUVECs. Cells were transfected with 10 nM AMPK siRNA or control siRNA (NC siRNA) for different times. **F.** and **G.** Visualization of tubulin depolymerization and actin polymerization in HUVECs. Cells were pretreated with 10 nM AMPK siRNA or NC siRNA for 24 h, followed by treated with 1 μM DPT for 30 min. **H.** G-LISA measurement of protein concentrations of GTP-bound RhoA in cell lysates of HUVECs. Cells were pretreated with 10 nM AMPK siRNA or NC siRNA for 24 h, followed by treated with 1 μM DPT for 30 min. Results are means ± SEM of at least three independent experiments. **p < 0.01, ***p < 0.001; ##*p* < 0.01, ###*p* < 0.001, compared with 0 h. Scale bar: 50 μm.

AMP-activated protein kinase (AMPK), a member of the MARK/PAR kinase subfamily (microtubule affinity-regulating kinase), has been recently recognized as a master molecular switch that promotes cytoskeletal remodeling. In the current study, we investigated the effects of the specific AMPK inhibitor compound C and AMPK siRNA on DPT-induced tubulin depolymerization and its downstream events. DPT strongly promoted the activation of AMPK in HUVECs. This effect was inhibited by the specific AMPK inhibitor compound C (Figure 3A). Interestingly, DPT-induced microtubule depolymerization and actin polymerization were reversed by compound C (Figure 3B and 3C). Additionally, this chemical inhibition also reversed RhoA activation mediated by DPT (Figure 3D). Consistent with the above results, AMPK protein levels were significantly decreased in HUVECs after transfection with AMPK siRNA for 24 h (Figure 3E–3H), demonstrating that DPT-mediated cytoskeletal remodeling is AMPK-dependent.

### LKB1-dependent AMPK activation is involved in DPT-mediated cytoskeletal remodeling in HUVECs

**Figure 4:**
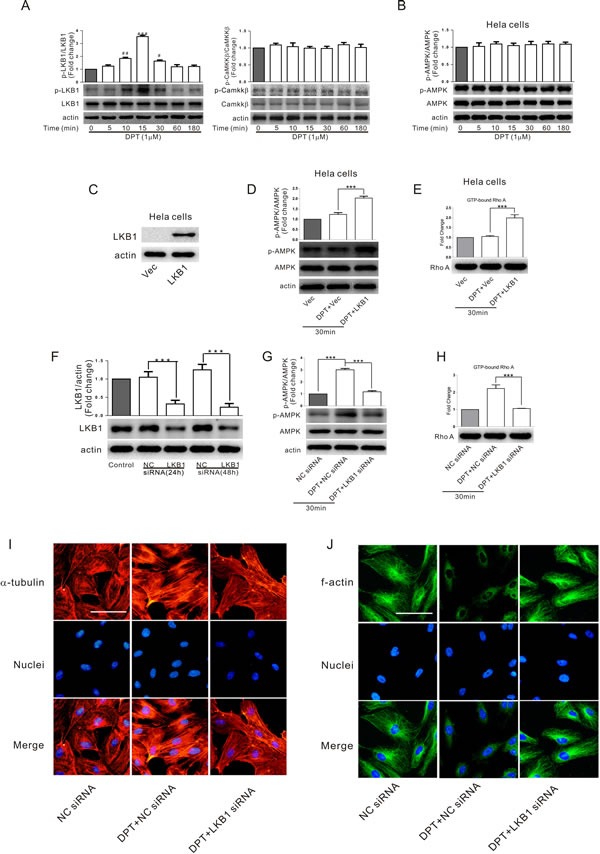
LKB1-dependent AMPK activation was involved in DPT-mediated cytoskeletal remodeling in HUVECs **A.** Western blot measurement of the expression levels of LKB1 phosphorylation and CaMKKβ phosphorylation in HUVECs. Cells were treated with 1μM Deoxypodophyllotoxin (DPT) for different times. **B.** Western blot measurement of protein expression of AMPK phosphorylation in LKB1-deficient Hela cells. Cells were treated with 1 μM DPT for different times. **C.** Western blot measurement of the expression levels of LKB1 in Hela cells. Cells were transfected with plasmid encoding for LKB1 gene or plasmid vector alone for 48 h. **D.** Western blot measurement of the expression levels of AMPK phosphorylation in Hela cells. Cells were pretreated with LKB1 plasmid or plasmid vector alone for 48 h, followed by treated with 1 μM DPT for 30 min. **E.** G-LISA measurement of protein concentrations of GTP-bound RhoA in cell lysates of Hela cells. Cells were pretreated with LKB1 plasmid or plasmid vector alone for 48 h, followed by treated with 1 μM DPT for 30 min. **F.** Western blot measurement of expression of LKB1 in HUVECs. Cells were transfected with 10 nM LKB1 siRNA or control siRNA (NC siRNA) for different times. **G.** Western blot measurement of the expression levels of AMPK phosphorylation in HUVECs. Cells were pretreated with 10 nM LKB1 siRNA or NC siRNA for 24 h, followed by treated with 1 μM DPT for 30 min. **H.** G-LISA measurement of protein concentrations of GTP-bound RhoA in cell lysates of HUVECs. Cells were pretreated with 10 nM LKB1 siRNA or NC siRNA or 24 h, followed by treated with 1 μM DPT for 30 min. **I.** and **J.** Visualization of actin polymerization and tubulin depolymerization in HUVECs. Cells were pretreated with 10 nM LKB1 siRNA or NC siRNA for 24 h, followed by treated with 1 μM DPT for 30 min. Results are means ± SEM of at least three independent experiments. ***p < 0.001; #*p* < 0.05, ##*p* < 0.01, ###*p* < 0.001, compared with 0 h. Scale bar: 50 μm.

Liver kinase B1 (LKB1) and calmodulin-dependent protein kinase kinase β (CaMKKβ) are upstream kinases which activate AMPK. In HUVECs, DPT activates LKB1 (as indicated by increased LKB1 phosphorylation) but not CaMKKβ (Figure 4A). Furthermore, AMPK was not activated by DPT treatment in LKB1-deficient HeLa cells (Figure 4B), indicating that LKB1 is the primary upstream kinase of AMPK in HUVECs. To further examine the potential role of LKB1 in AMPK-dependent RhoA activation, LKB1 was obviously expressed in Hela cells using plasmid transduction (HeLa LKB1). No LKB1 was detected in HeLa cells transfected with plasmid vector alone (HeLa Vec) (Figure 4C). RhoA and AMPK were significantly activated following DPT exposure in Hela LKB1 (Figure 4D and 4E). Consistently, LKB1 knockdown by siRNA in HUVECs markedly attenuated DPT-mediated AMPK phosphorylation, RhoA activation and cytoskeletal remodeling (Figure 4F–4J). However, pretreatment with the specific CaMKKβ inhibitor STO-609 did not prevent AMPK activation and cytoskeletal remodeling induced by DPT in HUVECs (Supplementary Figure 2). Altogether, these results indicate that LKB1 activation promoted AMPK-dependent cytoskeletal remodeling in HUVECs treated with DPT.

### DPT prevents intersegmental vessel formation in zebrafish via AMPK activation

**Figure 5:**
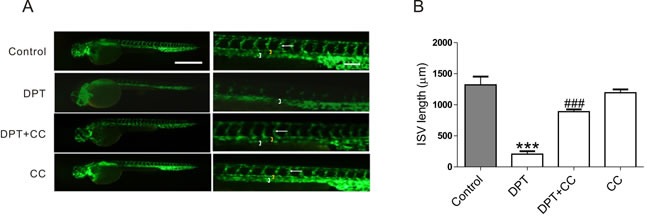
DPT-mediated intersegmental vessel (ISVs) defects in zebrafish through AMPK activation 24 hours old Transgenic zebrafish embryos were pretreated with 10 nM AMPK inhibitor Compound C (CC) for 2 h, followed by treatment with 50 nM Deoxypodophyllotoxin (DPT) for 24 h. **A.** The juvenile fish were narcotized and photographed by a ﬂuorescence stereomicroscope (SZX16; Olympus, Tokyo, Japan) at ×3.2 magnification (Scale bar: 500 μm) and ×10 magnification (Scale bar: 100 μm). Dorsal aorta (white bracket), posterior cardinal vein (yellow bracket) and ISV (white arrow) were highlighted. **B.** Statistical analysis of intersegmental vessel (ISVs) length. Results are means ± SEM. ***p < 0.001, compared with control; ###*p* < 0.001, compared with DPT.

Zebraﬁsh is a widely used model organism for studies of angiogenesis. The exposure of zebrafish embryos, which express green ﬂuorescent protein (GFP) in the developing vasculature, to DPT clearly caused blood vessel regression in the intersegmental vessel formation (ISVs) compared to control group. However, this effect was remarkably abolished by compound C which had no effect on the ISVs in zebrafish at the applied dosage in zebrafish (Figure 5).

### DPT exerts an anti-angiogenesis effect via AMPK activation in Matrigel plug assay

**Figure 6:**
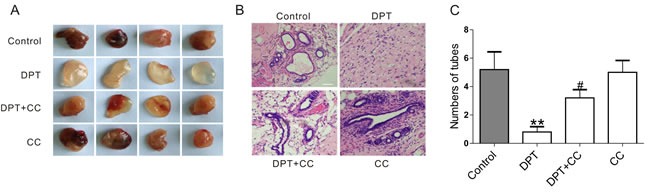
DPT-mediated anti-angiogenesis effect in Matrigel plug assay through AMPK activation Balb/c nude mice were administrated with 20 mg/kg Deoxypodophyllotoxin (DPT) for three times a week alone, or in the presence of 10 mg/kg Compound C (CC) for two weeks and sacriﬁced to obtain the matrigel plugs. **A.** The macroscopic appearance of Matrigel plugs from indicated groups. **B.** Staining of the Matrigel plugs with H&E, and evaluated by microscopy with a confocol microscope (FV-1000; Olympus, Tokyo, Japan). Scale bar: 50 μm. **C.** Statistical analysis of the numbers of vessels in 5 randomly areas of each slice. Results are means ± SEM. **p < 0.01, compared with control; # *p* < 0.05, compared with DPT.

We further evaluated the vascular disruptive effects of DPT *in vivo* using Matrigel plug assays. In mice receiving the vehicle control for two weeks, the plugs that were injected into the flanks of the mice became red, indicating the occurrence of vasifaction (Figure 6A). Integrated vascular lumen structures were observed by H&E staining (Figure 6B). However, plugs removed from DPT-treated mice were clear and pale, yellow in appearance, and no vascular lumen structures were observed. Notably, the vascular formation effects mediated by DPT were markedly reduced by compound C.

### DPT exerts anti-tumor and anti-vasculature effect via AMPK activation in xenograft mouse model

**Figure 7:**
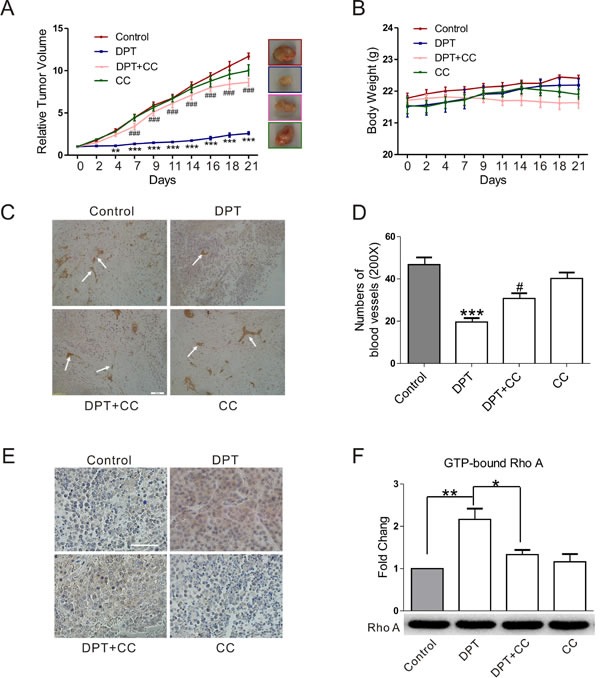
DPT-mediated anti-tumor and anti-angiogenesis effect in the xenograft mouse model The SGC-7901 xenograft mouse were randomly divided into 4 groups when the average tumor volume reached 100-160 mm^3^ and administrated with 20 mg/kg Deoxypodophyllotoxin (DPT) for three times a week alone, or in the presence of 10 mg/kg Compound C (CC) for three weeks and sacriﬁced to obtain the solid tumors. **A.** The effects of DPT on the relative tumor volumes of SGC-7901 xenograft mouse. **B.** The body weight of SGC-7901 xenograft mouse. **C.** and **E.** The effects of DPT on microvessel density (MVD) and p-AMPK phosphorylation of SGC-7901 xenograft mouse measured by immunohistochemistry and evaluated by microscopy with a confocol microscope (FV-1000; Olympus, Tokyo, Japan). Scale bar: 50 μm. **D.** Statistical analysis of the numbers of microvessels on five microscopic fields per specimen at ×200 magnification. **F.** G-LISA measurement of protein concentrations of GTP-bound RhoA in the lysates of solid tumors. Results are means ± SEM. * *p* < 0.015, **p < 0.01, ***p < 0.001 compared with control; # *p* < 0.05, ###*p* < 0.001, compared with DPT.

To confirm the effect of DPT on tumor growth and tumor vasculature, we examined its *in vivo* efficacy using SGC-7901 xenograft model, which is known to be sensitive to DPT therapy [10]. As depicted in Figure 7A, DPT treatment significantly suppressed tumor growth with an inhibition rate of 78% compared to control mice group (Figure 7A). Microvessel Density (MVD) of tumor sections was also dramatically decreased following DPT exposure (Figure 7C and 7D). No obviously damage was observed on the liver and gastric section of the host mouse (Supplementary Figure 3). Additionally, DPT did not affect mice body weight (Figure 7B) or cause other side effects. In contrast to DPT, compound C resulted in an obviously decrease of mice body weight at the end of the treatment-period. To further explore the *in vivo* molecular mechanism of DPT, p-AMPK staining and activated RhoA level were evaluated in solid tumors. Consistent with the results of the *in vitro* study, DPT caused significant increase of p-AMPK staining and RhoA activation (Figure 7E and 7F). Furthermore, compound C obviously reduced DPT-induced AMPK phosphorylation, RhoA activation, and tumor growth suppression, which is similar to the effects in the above experiment. These data confirmed that the *in vivo* inhibitory effects of DPT on tumor growth and tumor vasculature are mediated by AMPK-dependent RhoA activation.

## DISCUSSION

The present study was designed to determine whether DPT can act as a tumor vascular targeting agent by inducing cytoskeletal remodeling and to clarify the molecular mechanisms of such effect. The principal findings of our study are as follows: 1) DPT promotes cytoskeletal remodeling in HUVEC cells; 2) the molecular mechanism involved includes AMPK activation; and 3) LKB1 but not CaMKKβ is the upstream kinase involved in DPT-mediated AMPK activation.

Similarly to normal tissues, tumors require nourishment in the form of oxygen and nutrients as well as the ability to evacuate carbon dioxide and metabolic waste. The tumor vasculature generated by the process of angiogenesis addresses these needs [19]. The majority of tumors are unable to grow beyond a microscopic size of 1 to 2 mm^3^ without blood vessels [20, 21]. Therefore, targeting tumor vasculature is considered as a promising strategy to suppress tumor growth. More than 40 agents targeting tumor vasculature have been subjected to clinical trials [22, 23]. However, a lack of effect of angiogenesis inhibitors on existing tumor vessels shifted the focus to exploiting vascular-disrupting agents such as combretastatin A-4 phosphate which targets microtubules and is currently in phase I/II/III clinical trials [24].

DPT causes cytoskeletal remodeling and has both anti-angiogenic and vascular-disrupting activities at non-toxic doses [11]. Although RhoA/ROCK pathway has been shown to be implicated in DPT-induced suppression of tumor vasculature, the molecular mechanisms involved have not been fully characterized [11].

The development and functional maintenance of tumor vasculature involves the growth of new endothelial cells and their assembly into tubes (vasculogenesis) in addition to the sprouting (angiogenesis) of new vessels from existing ones [25]. Various components of the cytoskeleton (especially microtubules and microﬁlaments) play important roles in this process. Microﬁlaments are contractile structures consisting of actin molecules either in ﬁlamentous (F-actin) or monomeric (G-actin) forms. The monomeric status of the actin cytoskeleton facilitates endothelial cell migration [23]. DPT mediates an increase of F-actin and its subsequent assembly to form actin stress fibers accompanied by diffusion of microtubule network. DPT-mediated cell contraction was also observed through time-lapse imaging. Taken together, our results demonstrated that the anti-vascular effect of DPT results from its action on promoting cytoskeletal remodeling.

Endothelial cell contraction associated with stress fiber formation via Rho/Rho kinase pathway contributes to the vascular-disrupting activity [26]. Rho A activates the serine/threonine kinase ROCK which indirectly phosphorylates cytoskeletal proteins such as MLCs and cofilin and controls actin cytoskeletal contraction [27, 28]. In the present study, we investigated whether the RhoA/ROCK pathway regulates DPT-induced cytoskeletal remodeling. Our data revealed that DPT significantly increased the levels of GTP-bound RhoA (the active form of RhoA) in HUVECs. Additionally, time-dependent increases in MLC and cofilin phosphorylation were observed after DPT treatment. Treatment with either a ROCK antagonist or RhoA siRNA completely inhibited the effects of DPT on the actin cytoskeleton, indicating that mechanisms beyond RhoA activation contribute to the beneficial effects of DPT.

The loss of microtubules may result in the release of microtubule-associated proteins, thereby leading to the activation of RhoA [29]. Therefore, the possible relationship between tubulin and RhoA was also studied in this study. Incubation with taxol, a tubulin stabilizer, completely inhibited the effects of DPT on RhoA and actin. Consequently, the possible relationship between these proteins with regards to the effects of DPT on the cytoskeleton involves tubulin-RhoA/ROCK-actin. Despite the fact that overexpression of RhoA was reported in many cancer types, the actual role of RhoA in tumor progression depends on context [30]. In most cases, RhoA activation carries out the tumor vasculature suppressing effect of microtubule destabilizer when it is a downstream effector of microtubule depolymerization, consistent with our findings [3,11].

AMPK is a member of the MARK (microtubule afﬁnity-regulating kinase)/PAR kinase subfamily, which phosphorylates microtubule-associated proteins (MAPs) to inﬂuence microtubule dynamics and cell polarity [31–33]. Both synthetic and natural compounds that activate the AMPK pathway can promote cytoskeletal remodeling [18, 33–35]. However, investigations regarding the regulatory relationship between AMPK and the cytoskeleton are limited, and the mechanisms remain largely undefined. Moreover, it is unclear whether AMPK is a key regulator in tumor angiogenesis and whether the beneficial effect of DPT on promoting cytoskeletal remodeling is due to its activation of AMPK. We further investigated the mechanisms underlying DPT-promoted cytoskeletal remodeling. We observed that DPT robustly increased AMPK activation in HUVECs. Moreover, exposure to either an AMPK inhibitor or siRNA completely inhibited DPT-mediated tubulin depolymerization in HUVECs. However, actin polymerization is the downstream event of tubulin depolymerization and RhoA activation. The regulatory mechanisms among tubulin, Rho GTPases and actin are complicated. Actin polymerization sometimes are only in part through release of the depolymerized microtubule protein [29]. So AMPK siRNA seems to partially inhibit DPT-mediated actin formation. The *in vivo* zebrafish studies also validate DPT-mediated targeting tumor vasculature effects through enhanced AMPK activation [36]. DPT significantly inhibited intersegmental vessel formation (ISV) formation in transgenic zebrafish [Tg (VEGFR2:GFP)]. However, compound C (CC), a selective AMPK inhibitor, results in ISV defects at doses of 5 μM and 10 μM. Thus, we selected a dose of 10 nM, which partially dampens the DPT-mediated anti-angiogenesis effect in zebrafish. CC doses exceeding 100 nM exert synergistic anti-angiogenesis effects with DPT (data not shown). Interestingly, consistent with results obtained in zebrafish, DPT exhibited an inhibitory effect on vascular formation in the Matrigel plug assay, a proverbial model to assess tumor neovascularization [37] and in the xenograft mouse model. Moreover, no obviously damage was observed on the normal tissue sections. The selective effects of DPT on tumor vasculature perhaps result from the abnormalities of tumor vasculature [1]. Collectively, these results support the fact that DPT suppresses tumor vasculature through promoting cytoskeletal remodeling via AMPK signaling pathway both *in vitro* and *in vivo*.

AMPK is activated by the upstream AMPK kinases (AMPKK), LKB1 and CaMKKβ [38–41]. We investigated the effect of DPT on these upstream kinases and found that DPT promoted AMPK activation in HeLa cells transfected with plasmid encoding for LKB1. In addition, LKB1 siRNA blocked DPT-mediated AMPK activation in HUVECs, suggesting that LKB1 is the upstream kinase through which DPT activated AMPK. However, DPT significantly induced LKB1 and AMPK phosphorylation after 10-15 min exposure, while the tubulin destruction and F-actin formation elicited by DPT was observed at as early as 7.5 min. Cytoskeleton staining was therefore operated in HUVECs pretreated with AMPK siRNA for 24 h followed by DPT treatment for 7.5 min. As shown in Supplementary Figure 4, AMPK siRNA reversed DPT-mediated cytoskeletal remodeling. In contrast, the CaMKKβ pharmacological inhibitor STO-609 did not block the effect of DPT (Supplementary Figure 2), suggesting that DPT promoted cytoskeletal remodeling through the LKB1-AMPK pathway.

AMPK has been termed the fuel sensor of mammalian cells and its activation considered as tumor suppressor has been reported before ten years [42, 43]. However, the role of AMPK in cancer shows two faces [44]. Some reports show that AMPK is regulated by the tumor suppressor LKB1 and the tumor suppressors such as TSC2 and p53 are downstream effectors of AMPK. However, other reports show that AMPK may exert pro-tumor effects by regulating cellular metabolic plasticity [45, 46]. A positive or negative role for AMPK in tumor growth clearly depends on context. Moreover, AMPK-dependent cytoskeletal remodeling in tumor vasculature suppressing are few reported and whether it can be applied as a potential vascular targeting agent deserves further study.

A recent study reported that DPT inhibits the initiation of tubulin polymerization in a concentration-dependent manner as assessed by the extracellular tubulin polymerization turbidity assay, implicating that DPT directly binds to tubulin [47]. However, the reaction system of the extracellular tubulin polymerization turbidity assay is not identical to that in cells. Our results revealed that the effects of DPT on tubulin and RhoA/ROCK pathway were completely inhibited by either an AMPK inhibitor or AMPK siRNA. Thus, our data indicated that tubulin-RhoA is involved in DPT-induced cytoskeletal remodeling via downstream signaling of AMPK in HUVECs. Whether DPT directly binds to tubulin to cause it destruction remains to be investigated.

In conclusion, our studies demonstrated that DPT promotes cytoskeletal remodeling via activation of the LKB1-AMPK signaling pathway to suppress tumor vasculature both *in vivo* and *in vitro*. Due to the therapeutic effects and high safety profile, we expect that DPT can be used as a starting compound to develop novel therapeutic agents.

A new vascular network by angiogenesis is a key driver in tumor growth and metastasis [1, 22]. Nearly all of the anti-angiogenic agents currently in development or receiving FDA approval, preferentially target growth factor-induced cell proliferation and migration [2]. Little is known regarding the compounds that target the cytoskeleton changes of endothelial cells involved in angiogenesis. Noticeably, Microtubule-targeting agents (MTAs) thus far represent the only class of anticancer drugs that can be successfully used in anti-angiogenesis and vascular-disrupting strategies through cytoskeletal remodeling [3]. Moreover, we confirmed that DPT selectively targets tumor vasculature in xenograft tumor model. Thus, DPT can be exploited as a tumor vascular targeting agent to anti-tumor metastasis therapy.

**Figure 8:**
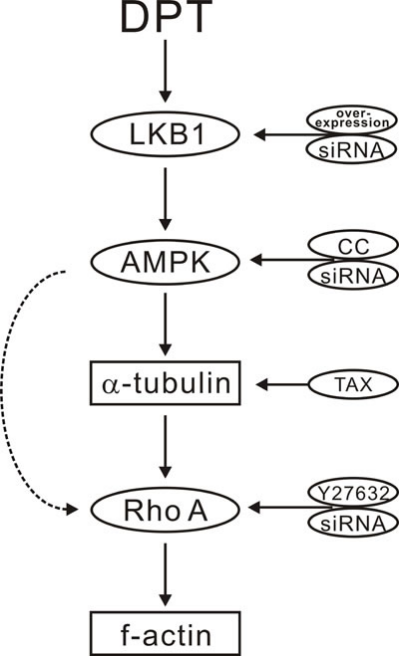
Scheme for the proposed mechanisms of deoxypodophyllotoxin (DPT) promoting cytoskeleton remodeling mediated by the LKB1-AMPK signaling pathway

## MATERIALS AND METHODS

### Ethics statement

This study was carried out in strict accordance with the recommendations in the Guide for the Care and Use of Laboratory Animals of the National Institutes of Health. Protocols described below were approved by the Southeast University Laboratory Animal Care and Use Committee. All surgery was performed under sodium pentobarbital anesthesia, and all efforts were made to minimize suffering.

### Reagents

DPT was obtained from the Medicinal and Chemical Institute, China Pharmaceutical University. Y27632, taxol, compound C, STO-609 were purchased from Sigma-Aldrich (St. Louis, USA). Anti-CD31, RhoA, MLC, p-MLC (Thr18/Ser19), coffilin, p-coffilin (Ser3), AMPK, p-AMPK (Thr172), LKB1, p-LKB1 (Ser428), CaMKKβ, p-CaMKKβ (Thr286) antibodies were purchased from Cell Signaling Technology (Beverly, MA). Anti-β-actin and α-tubulin antibody were purchased from Santa Cruz Biotechnology (CA, USA). Acti-stain555 Fluorescent Phalloidin was purchased from Cytoskeleton (Denver, USA).

### Cell culture

Human umbilical vein endothelial cells (HUVECs) were purchased from Sciencell (San Diego, USA). Each batch of HUVECs was examined using immunofluorescence and was shown to be CD31 positive (data not shown). Cells were cultured on plates coated with 0.5% collagen (Sigma-Aldrich, St. Louis, USA) in complete HUVECs medium supplemented with 10% fetal bovine serum (FBS, Gibco, Carlsbad, CA), 1% endothelial cell growth supplement, 100 U/ml penicillin and 100 μg/ml streptomycin (Sciencell, San Diego, USA). HeLa cells were cultured in DMEM (HyClone, Logan, UT), supplemented with 10% FBS, 100 U/ml penicillin and 100 μg/ml streptomycin. HUVECs (passage 3-5) and Hela cells that were approximately 70% confluent were seeded onto 6-well or 24-well plate for further experiments.

### Time-lapse microscopy

HUVECs were grown on collagen-coated 96-well glass-bottom dishes (*In Vitro* Scientific, Sunnyvale, CA) at 2×10^4^ cells/ml for 24 h at 37°C in 5% CO_2_ and then treated with either 1 μM DPT or 1 μM taxol. To collect time-lapse images, cells were observed every 2 min for 3 h using the IncuCyte Zoom (Essen, Ann Arbor, USA), a live cell imaging and data analysis system, in the incubator.

### Cytoskeleton immunoﬂuorescence

HUVECs were seeded at 2×10^4^ cells/ml on collagen-coated coverslips and cultured for 24 h. After treatment with DPT, cells were fixed with 4% paraformaldehyde for 30 min, permeabilized in 0.2% Triton X-100/phosphate-buffered saline (PBS) for 10 min, and blocked with 5% bovine serum albumin for 1 h to reduce nonspeciﬁc staining. The fixed cells were then incubated with an anti-α-tubulin primary antibody (4°C, overnight) followed by an Alexa Fluor 488-conjugated secondary antibody for 1 h. Filamentous actin was stained with Acti-stain 555 phalloidin and nucleus was stained with Hoechst 33342 for 10 min. Fluorescence images were obtained using a confocal microscope (FV-1000; Olympus, Tokyo, Japan).

### Western blot analysis

Cells were lysed in RIPA lysis buffer (Vazyme, Jiangsu, China). Cell homogenates were centrifuged at 13,200× rpm for 20 min. Total protein concentration of the supernatants was assessed by BCA kit (Thermo, Rockford, IL). Equal amounts of cell lysates were separated on 8%-15% SDS-polyacrylamide gel electrophoresis and electrophoretically transferred onto polyvinylidenedifluoride membranes (PVDF) (Millipore; Bedford, MA, USA). Membranes were then blocked with 5% BSA in Tris-Buffed-Saline with Tween (TBST) for 1 h, followed by incubation with diluted primary antibodies (overnight, 4°C). Membranes were washed with 0.1% Tween-20 in Tris-buffered saline (TBS) and incubated with horseradish peroxidase-conjugated secondary antibodies for 1 h at room temperature. The immunoreactive proteins were then detected by ECL-Plus Western Blotting Detection System.

### Measurement of activated RhoA by G-LISA

RhoA activity in HUVECs lysates and solid tumors were assayed using a commercial G-LISA kit (Cytoskeleton, Denver, USA) according to manufacturer specifications and read on a Molecular Devices M-5 microplate reader.

### RhoA, AMPK and LKB1 knockdown in HUVECs by siRNA

HUVECs cultured in collagen-coated 24-well plates were transfected with scrambled control siRNA, RhoA siRNA, AMPK siRNA or LKB1 siRNA (Genepharma, Shanghai, China) when cells reached 50% confluence. siRNA and RNA iMAX (Invitrogen, Camarillo, CA) were premixed in OPTI-medium (Invitrogen, Camarillo, CA) according to the manufacturer's instructions and then applied to cells. After 24 h transfection, OPTI-medium was replaced by complete HUVECs medium. Then HUVECs were treated with DPT for 30 min. All siRNA sense strands were listed in Table 1.

**Table 1:** Sequence of target gene siRNA

Gene	Sense strand (5′-3′)
AMPKα1 siRNA	GAGGAGAGCUAUUUGAUUATT
Rho A siRNA	CAGCCCUGAUAGUUUAGAATT
LKB1 siRNA	CCAACGUGAAGAAGGAAAUTT
Control siRNA	UUCUCCGAACGUGUCACGUTT

### LKB1 overexpression in Hela cells by plasmid transfection

HUVECs cultured in collagen coated 24-well plates were transfected with pcDNA 3.1(+) vector or pcDNA 3.1(+) encoding for LKB1 gene (Genepharma, Shanghai, China) when cells reached 30-40% confluence. Plasmid and Lipofectamine 2000 (Invitrogen, Camarillo, CA) were premixed in OPTI-medium (Invitrogen, Camarillo, CA) according to the manufacturer's instructions and then applied to the cells. After 48 h transfection, OPTI-medium was replaced by complete HUVECs medium. Then HUVECs were treated with DPT for 30 min.

### Effect of DPT on intersegmental vessel formation in zebrafish

Transgenic zebrafish that express green ﬂuorescent protein (GFP) in the developing vasculature [Tg (VEGFR2:GFP)] were maintained under standard conditions (Nusslein-Volhard and Dahm, 2002) [48]. Embryos were obtained through natural spawning and staged by time and morphological criteria [36]. DPT and compound C (CC) were diluted in dimethyl sulfoxide (DMSO) and then further diluted in fish water to the required concentrations. Experiments were performed in 24-well plates with 8 to 10 embryos per well in a volume of 1 ml. The chemical treatment was applied to 24-hour-old Tg (VEGFR2:GFP) embryos in fish water supplemented with either DMSO or CC. To characterize the intersegmental vessel (ISVs), embryos were dechorionated with 1 mg/ml of pronase before treatment. Embryos were narcotized and photographed 24 h after treatment.

### Matrigel plug assay

Female Balb/c nude mice (18 to 20 g) were purchased from Shanghai Rubicam Laboratory Animal Ltd. (Shanghai, China). Because DPT is not water-soluble, a 2-hydroxypropyl-β-cyclodextrin (HP-β-CD) inclusion complex (containing 3.06% of DPT) was prepared to further characterize its activity *in vivo*. The effects of DPT on angiogenesis *in vivo* were monitored using Matrigel plug assay. Prepared Matrigel (0.4 ml) containing 80 units/ml heparin (Sigma-Aldrich) and 100 ng/ml human recombinant VEGF-A_165_ (Peprotech, Rocky Hill, USA) was injected subcutaneously into the ﬂanks of mice. Animals were randomly divided into 4 groups and administered the following regimens thrice weekly: (a) HP-β-CD; (b) DPT (20 mg/kg) alone; (c) combination treatment of DPT (20 mg/kg) and compound C (10 mg/kg); and (d) compound C (10 mg/kg) alone. HP-β-CD, DPT, and compound C (Selleck Chemicals, Houston, USA) were all dissolved in normal saline. HP-β-CD and DPT were administered intravenously, while compound C was administered intraperitoneally. After 14 days, mice were sacriﬁced. Matrigel plugs were removed, ﬁxed in 10% neutral-buffered formalin, processed for embedding in parafﬁn, sectioned, and stained with hematoxylin and eosin (H&E).

### Mouse xenograft tumors study

Female Balb/c nude mice (18 to 20 g) were purchased from Shanghai Rubicam Laboratory Animal Ltd. (Shanghai, China). Viable SGC-7901 cells (3 × 10^6^/ 100 μl PBS per mouse) were subcutaneously (s.c.) injected into the right flank of the nude mice. When the average tumor volume reached 100-160 mm^3^, mice were randomly divided into 4 groups and treated as described in Matrigel plug assay. Tumor size and body weight were recorded three times a week with a caliper (calculated volume = shortest diameter^2^ ×longest diameter/2). After three weeks, mice were sacrificed and solid tumors were removed for further analyses.

### Immunohistochemistry

Solid tumors or normal tissues were fixed with 10% formaldehyde and embedded in paraffin. Blood vessel and p-AMPK staining were performed on 5 μm tumor sections with primary CD31 or p-AMPK antibody. Section images were obtained using a confocal microscope (FV-1000; Olympus, Tokyo, Japan). Microvessel density (MVD) in tumors was quantified as described previously [10].

### Statistical analysis

Statistical significance was determined using GraphPad Prism 5 software (San Diego, CA). Data are presented as mean ± SEM. Statistical analyses among multiple groups were performed using one-way ANOVA followed by Bonferroni's post hoc test. A *p* value less than 0.05 was considered to be statistically significant.

## SUPPLEMENTARY MATERIAL FIGURES


