# Supplementary material for: Deoxypodophyllotoxin suppresses tumor vasculature in HUVECs by promoting cytoskeleton remodeling through LKB1-AMPK dependent Rho A activation
Source: Oncotarget. 2015 Jul 22;6(30):29497–512. doi: 10.18632/oncotarget.4985 (PMC4745742; doi:10.18632/oncotarget.4985)
Supplement: Supplementary file 1 [file oncotarget-06-29497-s001.pdf]

Deoxypodophyllotoxin suppresses tumor vasculature in HUVECs by promoting cytoskeleton remodeling through LKB1-AMPK dependent Rho A activation

Supplementary Material

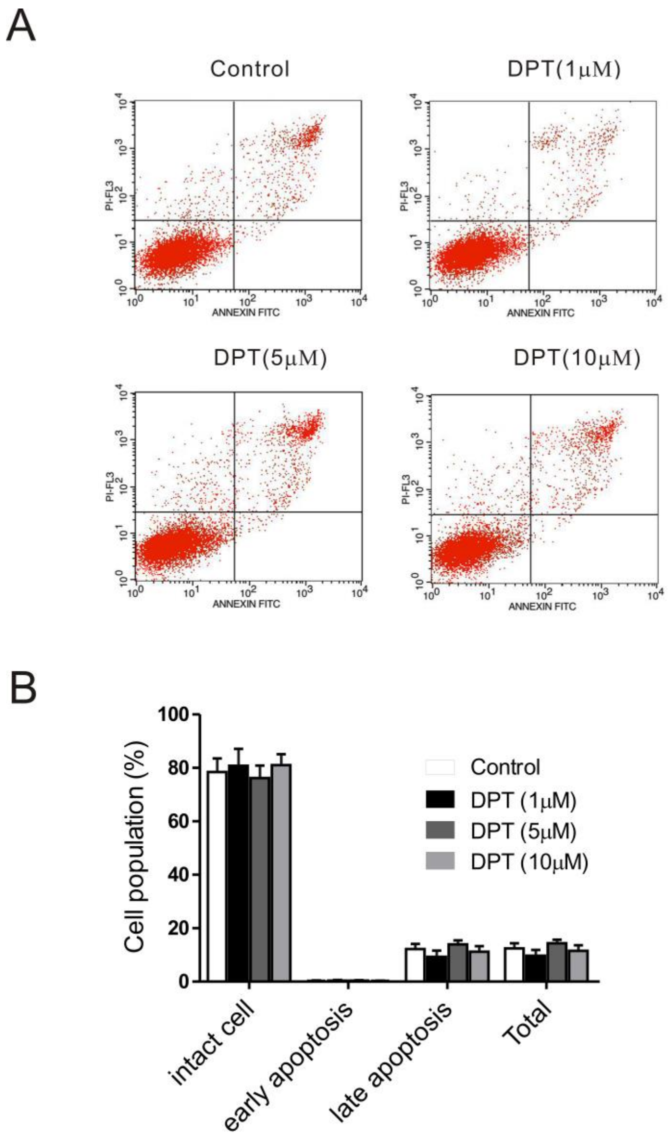

Supplementary Figure 1: DPT didn't induce apoptosis in HUVECs. Cells were treated with different concentrations of DPT for 3 h**. (A)** Annexin V-FITC/PI double-staining assay by flow cytometry. **(B)** Statistical analysis of the numbers of apoptotic cells. Results are means ± SEM of at least three independent experiments.

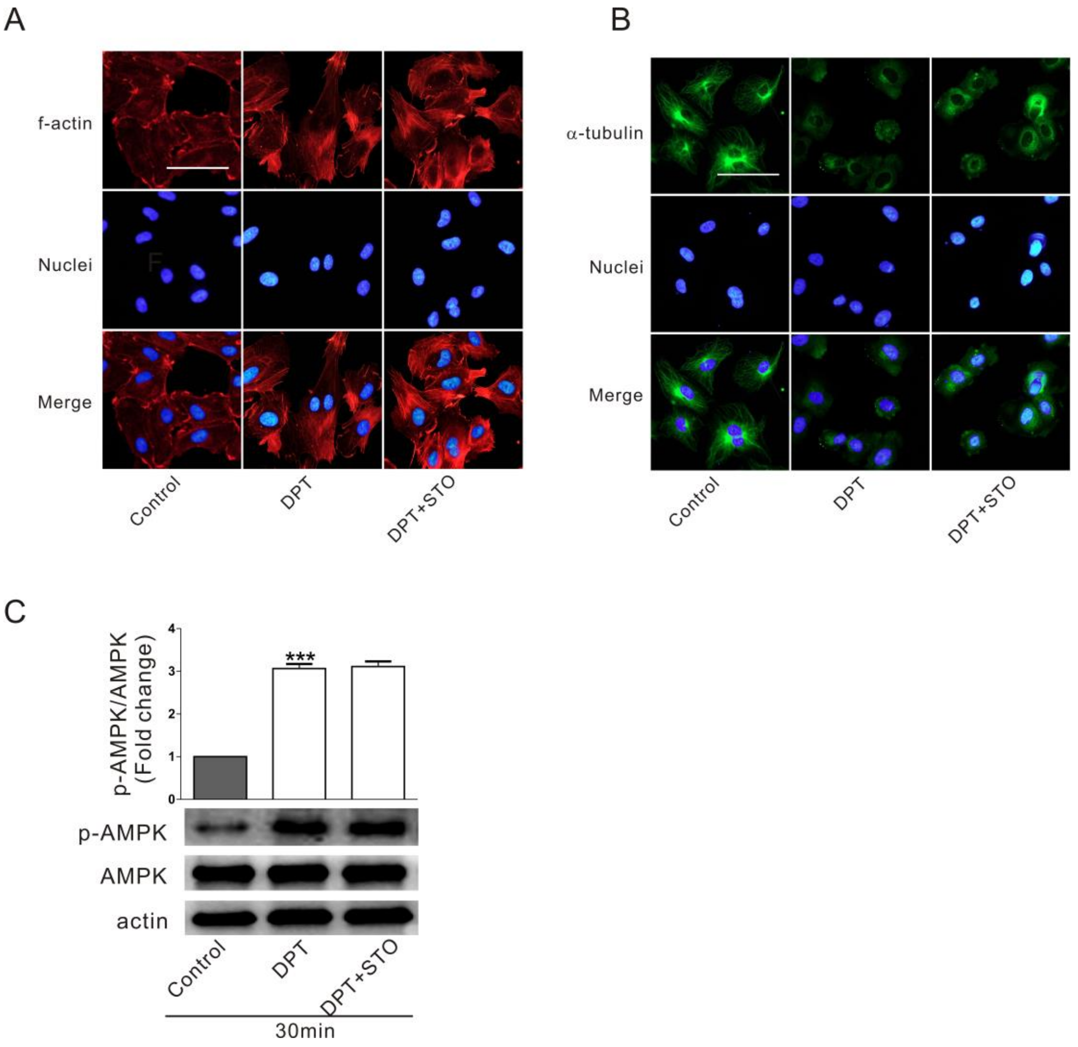

Supplementary Figure 2: Effects of CaMKKβ inhibitor on DPT-mediated AMPK phosphorylation and cytoskeletal remodeling. **(A)** and **(B)** Visualization of actin polymerization and tubulin depolymerization in HUVECs. Cells were pretreated with 20 μM CaMKKβ inhibitor STO-609 (STO) for 30 min, followed by treatment with 1 μM DPT for 30 min. Scale bar: 50 μm. **(C)** Western blot measurement of the expression of AMPK phosphorylation in HUVECs. Cells were pretreated with 20 μM CaMKKβ inhibitor STO for 30 min, followed by treatment with 1 μM DPT for 30 min. Results are means ± SEM of at least three independent experiments. ****p*<0.001, compared with Control.

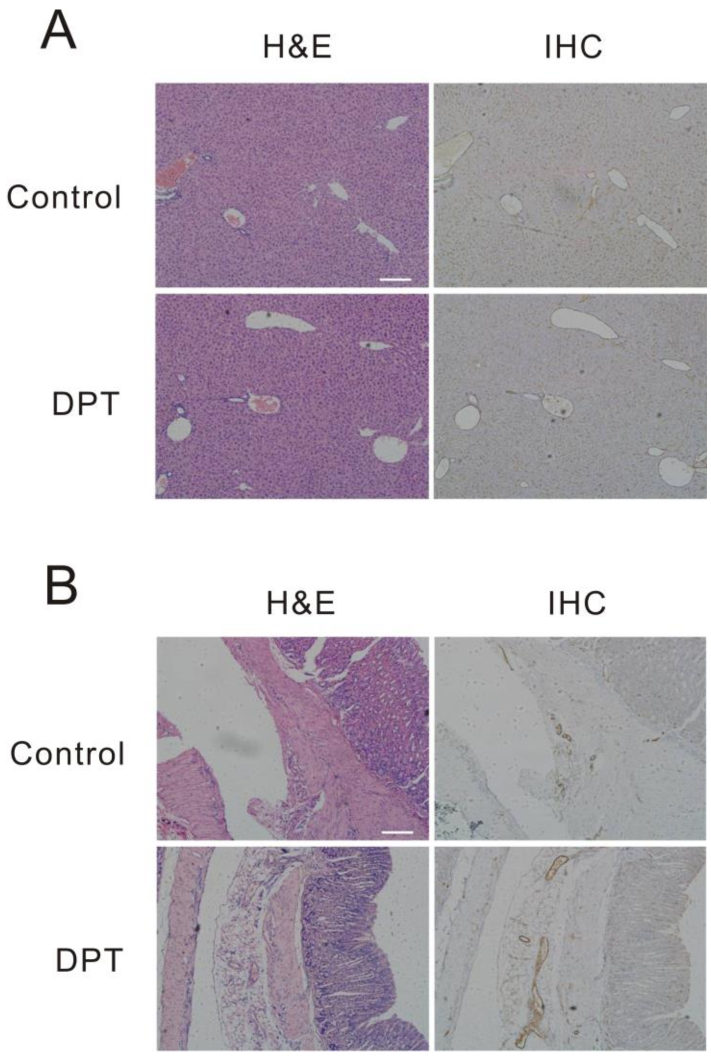

Supplementary Figure 3: Effects of DPT on normal vasculature. The SGC-7901 xenograft mouse were administrated with vehicle or 20 mg/kg DPT for three times a week when the average tumor volume reached 100-160 mm^3 for 21 days and sacrificed. Liver **(A)** and gastric **(B)** sections were stained by immunohistochemistry with a primary CD31 antibody and evaluated by microscopy with a confocol microscope (FV-1000; Olympus, Tokyo, Japan). Scale bar: 50 μm.

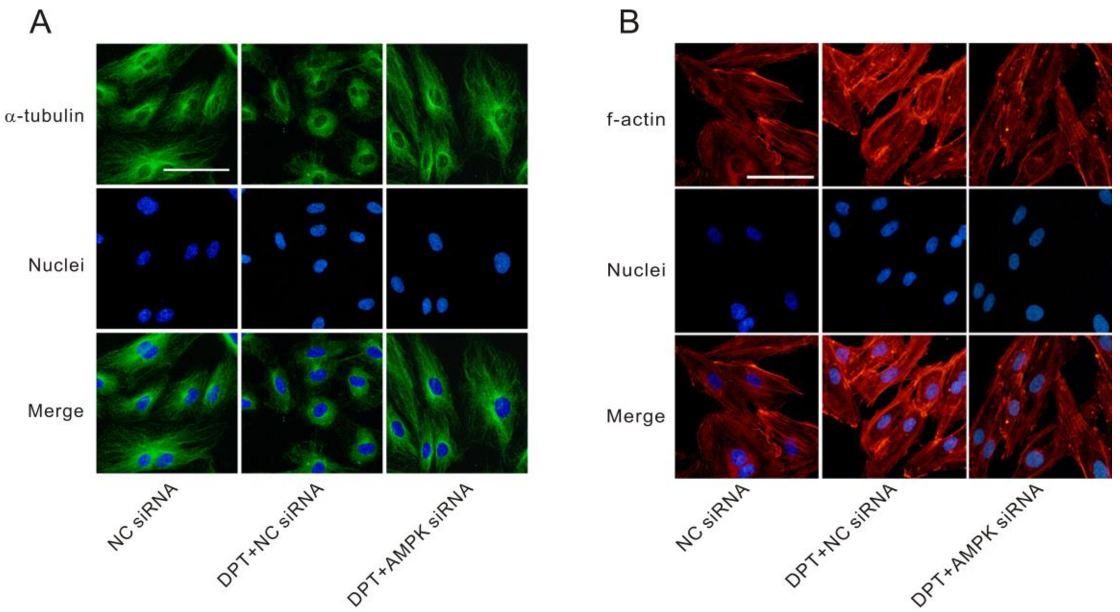

Supplementary Figure 4: AMPK activation was involved in DPT-mediated cytoskeletal remodeling in HUVECs. **(A)** and **(B)** Visualization of tubulin depolymerization and actin polymerization in HUVECs. Cells were pretreated with 10 nM AMPK siRNA or NC siRNA for 24 h, followed by treated with 1 μM DPT for 7.5 min. Scale bar: 50 μm.
